# Haplotype Reconstruction Error as a Classical Misclassification Problem: Introducing Sensitivity and Specificity as Error Measures

**DOI:** 10.1371/journal.pone.0001853

**Published:** 2008-03-26

**Authors:** Claudia Lamina, Friedhelm Bongardt, Helmut Küchenhoff, Iris M. Heid

**Affiliations:** 1 Institute of Epidemiology, Helmholtz Zentrum München – German Research Center for Environmental Health, Neuherberg, Germany; 2 Institute for Medical Information Processing, Biometry and Epidemiology, Ludwig-Maximilian University, Munich, Germany; 3 Institute for Statistics, Stablab, Ludwig-Maximilians University, Munich, Germany; University of Glasgow, United Kingdom

## Abstract

**Background:**

Statistically reconstructing haplotypes from single nucleotide polymorphism (SNP) genotypes, can lead to falsely classified haplotypes. This can be an issue when interpreting haplotype association results or when selecting subjects with certain haplotypes for subsequent functional studies. It was our aim to quantify haplotype reconstruction error and to provide tools for it.

**Methods and Results:**

By numerous simulation scenarios, we systematically investigated several error measures, including discrepancy, error rate, and R^2^, and introduced the sensitivity and specificity to this context. We exemplified several measures in the KORA study, a large population-based study from Southern Germany. We find that the specificity is slightly reduced only for common haplotypes, while the sensitivity was decreased for some, but not all rare haplotypes. The overall error rate was generally increasing with increasing number of loci, increasing minor allele frequency of SNPs, decreasing correlation between the alleles and increasing ambiguity.

**Conclusions:**

We conclude that, with the analytical approach presented here, haplotype-specific error measures can be computed to gain insight into the haplotype uncertainty. This method provides the information, if a specific risk haplotype can be expected to be reconstructed with rather no or high misclassification and thus on the magnitude of expected bias in association estimates. We also illustrate that sensitivity and specificity separate two dimensions of the haplotype reconstruction error, which completely describe the misclassification matrix and thus provide the prerequisite for methods accounting for misclassification.

## Introduction

Haplotypes have been the subject of considerable attention as they complement the information from the SNP (single nucleotide polymorphism) genotypes. When viewing the nucleotides of the two DNA strands as two long rows of code, we consider haplotypes to summarize the horizontal multi-locus information per strand while genotypes summarize the vertical information on a single locus across the two strands. There are several advantages of haplotypes: In regions of high linkage disequilibrium (LD), haplotype diversity is said to be limited resulting in only a few existing haplotypes and thus a gain in power for the analyses [1], [2]. Multilocus haplotypes may capture the LD information in a gene better than methods based on single loci [3]. Furthermore, latent functional loci may be identified by haplotypes as they serve as multiallelic markers. Finally, the haplotype may represent the biologically functional genetic unit rather than the genotypes [4]. Haplotypes can thus provide additional information with respect to association analysis and localization of complex disease genes [5], especially in the presence of multiple susceptibility alleles [6].

One draw-back of haplotypes is the fact that experimental derivation of haplotypes is still not practical for the large number of individuals in epidemiological studies, but has to be inferred statistically. The most frequently used methods for haplotype reconstruction are based either on the maximum likelihood-based expectation-maximization (EM) algorithm [7] or on a Bayesian framework incorporating the coalescent model [8], [9]. The performance of the various reconstruction methods have already been compared by some groups on real or simulated datasets [8]–[12]. PHASE, for example, has been found to outperform other methods, if the data were generated by a coalescent model [8]. But clearly all methods involve a certain amount of error in the reconstructed haplotypes.

Fallin and Schork [13] investigated the haplotype error using the mean squared error (MSE), which was found to increase with increasing minor allele frequency (MAF), decreasing LD and increasing number of loci. However, the MSE summarizes the error in the estimated haplotype frequencies rather than the error in the individuals' haplotypes, which is of interest in haplotype association analyses. One commonly used error measure for the error in the individuals' haplotypes is the error rate of which some authors have described selected aspects [8], [10]–[12] . However, a systematic investigation of the error rate is still lacking. Furthermore, researchers are often interested in subjects carrying a specific haplotype and thus rather in the error in assigning this haplotype than in an error measure averaging across all haplotypes. Individual haplotypes are coded as trichotomous variables, which results in a straight-forward 3×3 misclassification problem. In association analysis models, misclassification in independent variables, in this case the haplotypes, are known to yield biased estimates [14], [15]. The amount of bias depends on the magnitude of the error. If the corresponding 3×3 misclassification matrix is known, it completely describes the error mechanism and can be used for post-hoc adjustment of haplotype association analyses.

There are methods available, which do not infer individual haplotypes but instead use estimates of the distribution of haplotypes given the observed genotypes to estimate risk parameters within a likelihood framework ([16]–[21]). However, these methods are often limited to specific study types or modelling techniques. Inferred individual haplotypes provide the advantage of being easily adapted into the framework of generalized linear models and can thus be analyzed with standard statistical software. For haplotypes that are fairly well reconstructed, the analysis of inferred haplotypes is unbiased and is more powerful than the analysis based on expected haplotype probabilities [22]. For haplotypes with higher uncertainty in reconstruction, information of the misclassification probabilities can be used to correct association estimates, for example using the MC-SIMEX method [23].

However, downstream association analyses are not the only reason for inferring haplotypes. Haplotypes can also be used to put up phylogenetic trees or for selection of certain subjects in a study. Researchers in the need to select study subjects with specific haplotypes for subsequent in-depth functional studies use the haplotype assignment to individuals and rely on sufficient assignment certainty for subject selection.

For these reasons we think it is worthwhile to have a closer look at haplotype uncertainty with special emphasis on misclassification probabilities of individually inferred haplotypes.

Therefore, we aimed at a general understanding of the magnitude of haplotype reconstruction error in a systematic approach by simulations and analytical derivations. We applied various simulation scenarios including scenarios based on realistic haplotype distributions from our epidemiological study at hand. We present a systematic overview of error measures with focus on haplotype-specific error measures posing a classical misclassification problem and present the sensitivity and specificity as two intuitive measures. We outline analytical and simulation approaches to quantify several error measures, describe the size and dependencies of these haplotype errors and show the impact on odds ratio estimates.

## Methods

### Notation and Definitions

Let *L* be the *number of loci* and *N* the *number of individuals*. For each individual *i* = 1, …,*N*, the vector *G_i_* = (*G_i1_*,…,*G_iL_*) denotes the subject's *genotypes* at the *L* loci, *l* = 1, …*L*, with *G_il_* indicating the number of minor alleles at locus *l* for individual *i* and *G_il_*∈{0,1,2}. Consequently, there are 3*^L^* possible values γ = (γ*_1_*,…,γ*_L_*) for *G_i_*. There are *M* = 2*^L^* possible different haplotypes *h*_1_, …, *h_M_* in the population, the haplotypes of subject *i* can be written as a vector *H_i_* = (*H_i1_*,…,*H_iM_*), with each *H_im_* indicating *the true number of copies of the haplotype h_m_* of subject *i*, *m* = 1, …, *M*, and *H_im_*∈{0,1,2}. Due to the restriction 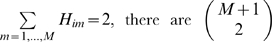 possible values η = (η_1_,…, η*_M_*) for *H_i_*. *H_i_* thus denotes the individual's haplotype pair (“diplotype”) and the various η reflect all possible pairs. The number of different pairs actually appearing in a sample is further restricted by the correlation between the alleles at the loci. The effective number of loci, *L_eff_*, can be computed according to Nyholt [24] taking this correlation into account. Note that the term “allele” and “haplotype” is not used completely consistent in the literature. We define an *allele* to be one of the different values on one chromosome at a SNP locus and haplotypes to be the combination of the alleles across several loci. The diploid human beings thus exhibit two alleles (at one SNP locus) and two haplotypes (across several SNP loci).

When statistically reconstructing haplotypes from genotypes, the *reconstructed number of copies of each haplotype* in subject *i* is denoted as H_i_* = (H_i1_*,…,H_iM_*) being the vector of the expected values given the observed genotypes *G_i_* as estimated by a reconstruction program: H_i_* = *E*(*H_i_*|*G_i_*). As an unambiguous decision for a haplotype pair is not always possible, the H_im_* move in a continuous space, H_im_* ∈ [0,2]. The most probable or most likely number of haplotypes C_im_* is derived by categorizing H_im_* into the most likely haplotype pair for each individual with C_im_* indicating *the observed number of copies of the haplotype h_m_*, thus returning to the discrete space, C_im_*∈{0,1,2}. They are also often denoted as individually inferred haplotypes.

Haplotypes can be inferred unambiguously (i.e. without error) for subjects being heterozygous in less than two loci. The *ambiguity fraction* is the number of subjects being heterozygous for at least two loci, *N_amb_*, divided by *N*, which thus describes the proportion of the sample where haplotype reconstruction error might occur.

The *frequencies* of the haplotypes *h*_1_,…, *h_M_* in the sample are denoted as *f* = (*f*_1_,…, *f_M_*) with 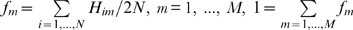. The sampling error for estimating the frequency is considered to be ignorable in large enough data sets. Analogously, the frequencies of the reconstructed haplotypes H_i_* are denoted as f* = (*f*_1_*,…,f_M_*).

### Measures of the haplotype reconstruction error

The accuracy of haplotype reconstruction can be measured in different ways for different purposes. We propose a classification based on three characteristics: (1) The uncertainty across all haplotypes (1a, “overall error measure”), versus the error in a specific haplotype (1b, “haplotype-specific error measure”). (2) The uncertainty in a sample statistics (2a, i.e.: haplotype frequencies, *f*→f*) versus the uncertainty in individuals' haplotypes (2b). (3) To further differentiate 2b: The error made by using the expected number of haplotype copies, *H*→H* (3a), versus the error made by using the most probable haplotype, *H*→C* (3b). The measures are defined and related to the above stated classes in the following (for a summary, see Table 1):

**Table 1:** Classification of measures for the haplotype reconstruction error.

	*Error in haplotype frequency*	*Error in subject-specific haplotype H_i_* §
*Overall measure*	Discrepancy (D)	Error rate among all subjects (ER_all_)
		Error rate among ambiguous subjects (ER_amb_)
*Haplotype-specific measure*	Discrepancy per haplotype (D_m_)	Correlation between true and reconstructed haplotypes (R_m_^2^)
		Sensitivity (Sn_m_) and Specificity (Sp_m_)
		Misclassification probabilities

§ *H_i_* denotes the vector of length *M* coding the number of copies of true haplotypes of subject *i*, *i* = 1, …, *N* for the *m* = 1, …, *M* possible haplotypes.

#### Discrepancy

The discrepancy *D* is the average of the differences between true and reconstructed haplotype frequencies, providing an “overall measure” of the error *f*→f* based on the summary statistics *f* instead of the subjects' haplotypes (*class 1a*, *2a*):
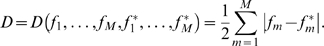
A haplotype-specific discrepancy is given by 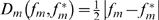 for *m* = 1,…, *M* (*class 1b*, *2a*). The discrepancy is close to the mean squared error (MSE) [11], [13], which is another way of averaging.

#### Error rate

The error rate among all individuals,
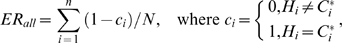
is the proportion of subjects with falsely classified haplotypes. Another definition is the error rate restricted to the subjects with ambiguous reconstruction, *ER_amb_*, where *N_amb_* replaces the N in the denominator [8]. *ER_all_* and *ER_amb_* are “overall measures” of the error *H*→C* (*class 1a*, *2b*, *3b*).

#### Proportion of explained variance, *R^2^_m_*

*R_m_^2^*, defined as the squared correlation between *H_im_* and H_im_* [25], *m* = 1, …, *M*, is a haplotype-specific measure for the error *H*→H* (*class 1b*, *2b*, *3a*). It is computed as the ratio of the haplotype variance explained by the genotypes, Var(H_im_*), to the variance of the *Bin*(2, *f_m_*)-distributed (true haplotype frequency), 2*f̂* _m_*(1−*f̂* _m_*), assuming no error in the haplotype frequency from reconstruction (*f_m_* = f_m_*).

#### Sensitivity and specificity

In the context of haplotypes, sensitivity and specificity are defined as “the probability that a true carrier of a certain haplotype is classified as such” (*sensitivity*) and “the probability that a true non-carrier is classified as such” (*specificity*), respectively, for *m* = 1, ‥, *M*,
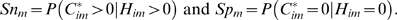
Thus, 1−*Sn_m_* and 1−*Sp_m_* measure the “haplotype-specific error” *H*→C* (*1b*,*2b*,*3b*).

#### Misclassification probabilities

The error resulting from the transition *H*→C* is a pure misclassification problem for a trichotomous variable, which is described by a 3×3 misclassification matrix consisting of the misclassification probabilities π*_kl_* = *P*(C_im_* = *k*|*H_im_* = *l*), *k*,*l* = 0,1,2. Assuming no genotyping error, the subjects truly having two copies of a haplotype (true homozygous) as well as subjects with two copies of a haplotype in the reconstruction (observed homozygous) have always homozygous genotypes for all loci. These haplotypes can be reconstructed unambiguously and the misclassification probabilities π_20_, π*_21_*, π*_02_*, and π*_12_* equal zero. The misclassification matrix is then completely determined by sensitivity, specificity and the true haplotype probabilities or the observed haplotype probabilities (see Table 2).

**Table 2:** Misclassification matrices for haplotype reconstruction error.

*Misclassification matrix in its most general form*					
		Reconstructed C_im_*			
		0	1	2	∑
**True** *H_im_*	**0**	π_00_^(*m*)^	π_10_^(*m*)^	π_20_^(*m*)^	**1**
	**1**	π_01_^(*m*)^	π_11_^(*m*)^	π_21_^(*m*)^	**1**
	**2**	π_02_^(*m*)^	π_12_^(*m*)^	π_22_^(*m*)^	**1**

Misclassification probabilities: π*_kl_* ^(*m*)^ = *P*(C_im_* = *K*|*H_im_* = *l*), *k*,*l* = 0,1,2, *m* = 1,…, *M*, for subject *i*; sensitivity: *Sn_m_* = *P*(C_im_*>0|*H_im_*>0); specificity: *Sp_m_* = *P*(C_im_* = 0|*H_im_* = 0); true or reconstructed haplotypes' probabilities (i.e. probabilities that a subjects has *k* number of copies of haplotype *h_m_*): π*_k_* ^(*m*)^ = *P*(*H_im_* = *k*), *k* = 0,1,2; π*_k_* ^(*m*)^* = *P*(C_im_* = *k*); true or reconstructed number of copies of *h_m_*: *H_im_* or C_im_*, respectively.

### Data

SNP data on numerous genes in a subsample of the population-based KORA study were available as examples. This sample of 704 individuals aged 55 to 74 years was a subset of the fourth survey (S4) of the KORA (Cooperative Research in the Region of Augsburg) study from 1999–2001 [26]. Genotypes were obtained via mass spectrometry (MALDI-TOF MS). The 8 genes in this investigation had been discussed as possible risk factors for diabetes, but had shown no or only a small association [27], [28] : *IL-18*, *IL-13*, *MIP1A* , *INS*, *IL-6*, *MCP1*, *TNFA*, and *CAPN10*. We reconstructed haplotypes by the EM algorithm and derived haplotype frequencies. Depending on the gene, 2–7 loci were involved. Additionally, haplotype frequencies were derived by PHASE, but the differences to EM-derived haplotype frequencies were very small and had no impact on our results, and thus results using PHASE-derived input haplotype frequencies are not reported here.

### Simulations and analytical approach to quantify haplotype reconstruction error

In the simulations, true haplotype frequencies were taken as input parameters. For each simulation run, 1000 haplotypes were randomly drawn given the haplotype frequency distribution thus creating two copies of the haplotypes for 500 subjects assuming Hardy-Weinberg equilibrium. Genotypes were deduced and haplotypes were reconstructed from these genotypes using the EM as well as the PHASE algorithm. The reconstructed haplotypes were compared with the true haplotypes using the various error measures. For 100 simulations, the mean and the standard deviation of the error measures were computed. These mean error measures from the simulations were compared with analytical computations, which we derived (Appendix S1).

To derive the true haplotype frequencies as input parameters, different scenarios were implemented for the simulations and the analytical computations:

*Abstract scenarios* included three types: (*a*) A two-locus scenario varying the frequency *f_1_* of haplotype *h_1_*, while two other frequencies *f_3_* and *f_4_* are set at 0.1 and 0.05. (*b*) Another two-locus scenario varying the MAFs of locus 1 and locus 2, *MAF_1_* and *MAF_2_*, and the correlation r. With 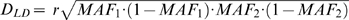 [29], the haplotype frequencies were derived as *f*_1_ = *D_LD_*−*MAF*_1_·*MAF*_2_, *f*_2_ = −*D_LD_*+(1−*MAF*_1_)·*MAF*_2_, *f*_3_ = −*D_LD_*+*MAF*_1_·(1−*MAF*_2_), and *f*_4_ = *D_LD_*−(1−*MAF*_1_)·(1−*MAF*_2_). (*c*) Various multi-locus scenarios assuming equal MAFs for 3–6 loci under the assumption of *r* = 0.

*Real data scenarios* used the sets of haplotype frequencies as they were observed in the KORA data described above.

### Impact of haplotype reconstruction error on odds ratio estimates

To evaluate the impact of haplotype misclassification on association estimates, we constructed a case-control study with 500 controls and 500 cases, with a given “true” Odds Ratio (OR) and frequency of the risk haplotype assuming a dominant genetic model. With these specifications, a simple 2×2 contingency table can be constructed. Via the matrix method [30], we derived an analogous contingency table that could be expected to be observed under the given sensitivity and specificity specifications. From these, we can calculate the “observed” OR and compare it with the given OR.

## Results

### Discrepancy

**Figure 1:**
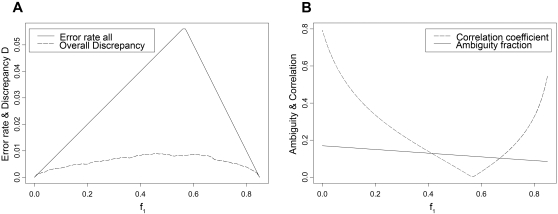
Discrepancy and error rate depending on haplotype frequency: A) Discrepancy (from simulations) and error rate (analytically derived), B) ambiguity fraction and correlation coefficient r (Abstract type a scenarios: two loci varying frequency f_1_ of haplotype h_1_ = 00 with f_3_  = 0.1 and f_4_  = 0.05 for h_3_ = 01 and h_4_ = 11).

Figure 1A illustrates that the discrepancy increases steadily with increasing frequency of one haplotype (other frequencies fixed, *abstract scenarios type a*) until it reaches a maximum of 0.00917 for *f_1_* = 0.46, and then, for *f_1_*>0.61, it decreases monotonically. The discrepancies in *real data scenarios* (Table 3) show values below 0.005 indicating an average difference between true and reconstructed haplotype frequencies below 0.5%, except for *MCP1* and *CAPN10*. There is a small difference when comparing EM- or PHASE-derived haplotypes yielding a smaller discrepancy using the EM for *INS* and *MCP1*, and a smaller discrepancy using PHASE for *IL-6*.

**Table 3:** Discrepancy for real data scenarios.

*l_eff_*	*l*	*Gene*	*EM*	*PHASE*
1.02	2	*IL-18*	0.0000±0.0000	0.0000±0.0000
1.06	3	*IL-13*	0.0001±0.0003	0.0001±0.0003
1.15	2	*MIP1A*	0.0002±0.0004	0.0002±0.0004
1.69	4	*INS*	0.0003±0.0007	0.0005±0.0006
2.31	3	*IL-6*	0.0008±0.0011	0.0006±0.0007
2.96	3	*MCP1*	0.0130±0.0070	0.0150±0.0080
3.00	3	*TNFA*	0.0040±0.0030	0.0040±0.0030
6.38	7	*CAPN10*	0.0320±0.0090	0.0320±0.0100

Values given are discrepancy D±standard deviation using the EM- or PHASE-reconstruction, stating the number of effective loci, *l_eff_*, and the number of loci, *l*.

### Error rate

For the two-locus scenario varying one haplotype frequency (*abstract scenarios type a*), the error rate (see Figure 1A) reaches a maximum of 0.056 for *f_1_* = 0.57 and is minimal for small *f_1_* or for large *f_1_*. To understand the location of this maximum, the ambiguity and the correlation coefficient *r* are displayed in Figure 1B: At the maximum, the alleles show zero correlation. It also becomes apparent that the correlation has a stronger influence on the error rate than the ambiguity in this 2-locus case.

**Figure 2:**
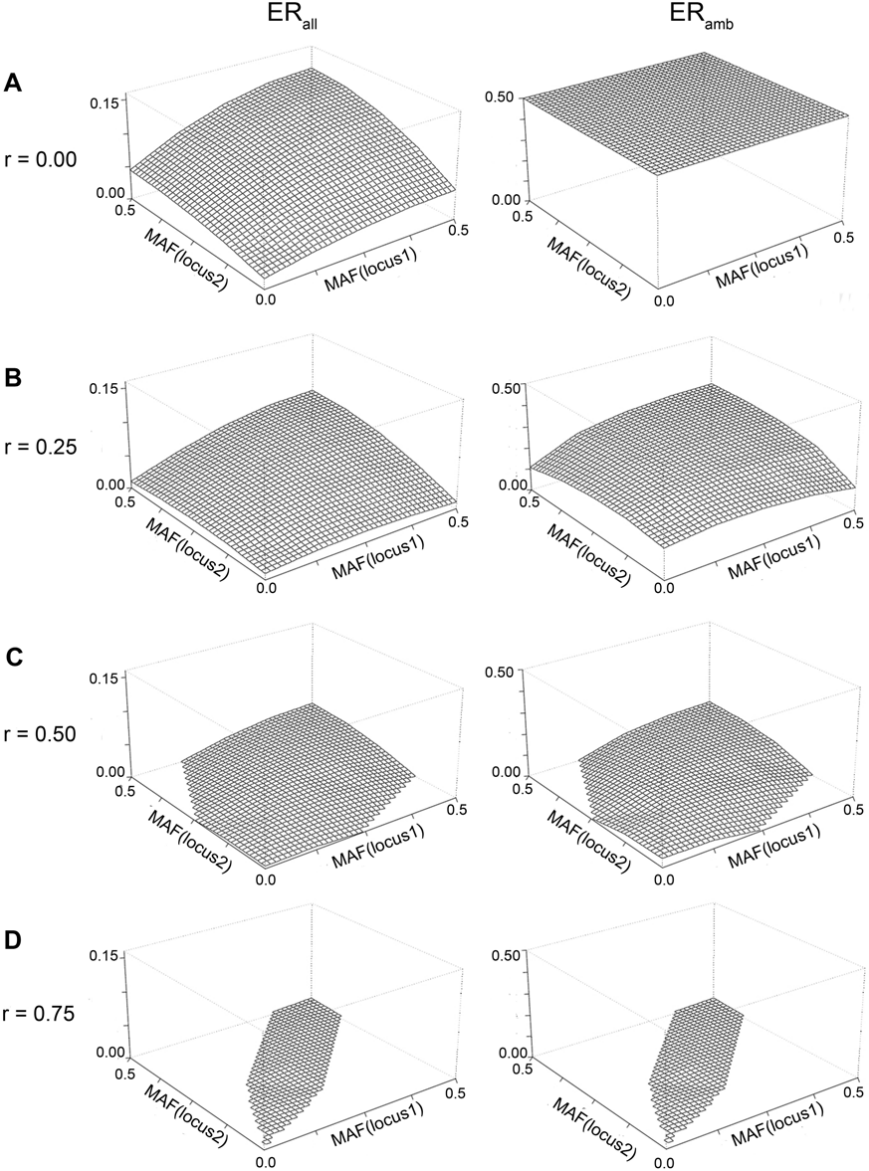
Error rate for varying MAF and correlations for two loci: Analytically derived error rate for A) r  =  0, B) r  =  0.25, C) r  =  0.5, D) r  =  0.75 (Abstract type b scenarios)

Figure 2 depicts the dependency of the error rate on the MAFs and r (*abstract scenario type b*): the smaller the MAF, the smaller the error. The error is minimal, when both MAFs are small. This is due to the fact that the ambiguity is smaller for lower MAF as there are fewer genotypes deviating from the wildtype and thus fewer heterozygotes. Furthermore, the error decreases for increasing *r*. Note that high correlation imposes strong restrictions on the possible MAF combinations, as the MAF of the second locus can only slightly deviate from the MAF of the first locus and thus the parameter space is reduced (Figure 2D). When *r* = 0, *ER_amb_* is 0.5, which is like flipping a coin (Figure 2A) for assigning haplotypes to ambiguous subjects.

**Figure 3:**
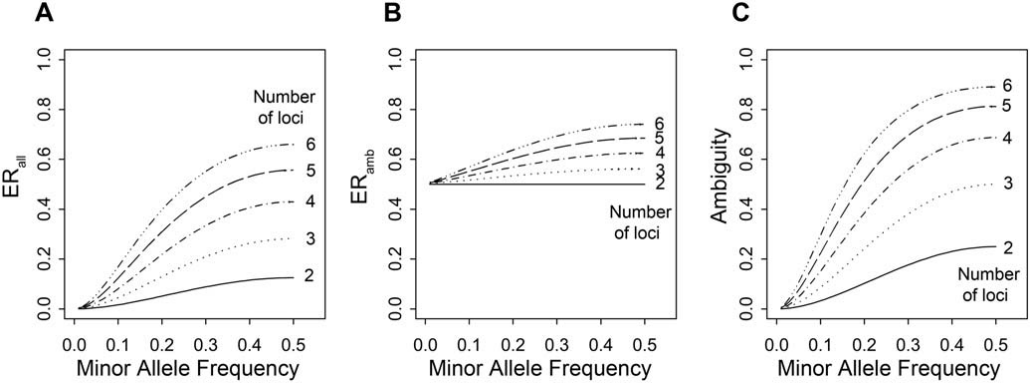
Error rate for varying number of loci and MAF under no correlation: A) ER_all_ and B) ER_amb_ and C) ambiguity analytically derived for abstract type c scenarios (2-6 loci, r = 0, equal MAF at each locus).

In Figure 3A, which depicts the multi-locus scenarios under no LD (*abstract scenarios type c*), it can be seen that the error rate increases with the number of loci. This is due to the fact that the probability of a subject being heterozygous in at least two loci increases with the number of loci involved, which is depicted by the increasing ambiguity fraction (Figure 3C). But this is not the sole reason as it can be seen that also *ER_amb_* increases with the number of loci (Figure 3B): The number of haplotypes increases and thus the pool for misclassification enlarges. It can further be seen, that in the case of MAF = 0.5 when all alleles and consequently all haplotypes are equally frequent, the *ER_amb_* is as large as when a die was rolled for haplotype assignment of ambiguous subjects. This is due to the fact that then the haploptype inference is guided by neither the correlation nor the haplotype frequency. When MAF<0.5 and haplotypes occur with different frequencies, the reconstruction can improve by preferring haplotype pairs containing more frequent haplotypes. Note that Figure 3 shows a worst-case scenario indicating the maximum possible error due to the no-LD assumption. As it is unreasonable to infer haplotypes in such a situation in the first place, these error rates remain unmatched in real data scenarios. It should further be noted that the error rate derived from simulations instead of using the analytical approach was practically the same, but slightly lower (data not shown).

Table 4 shows that the error rates for real data scenarios vary substantially between genes: The error rate is large for genes with low LD between loci, which are the genes showing a small difference between the number of loci and the effective number of loci (e.g. for *MCP1*, *TNF*α, *CAPN10*). For most genes, the error rate is well below 1%, which indicates that for 99% of the subjects the haplotypes are perfectly reconstructed. In these real data scenarios, the error rate of PHASE-reconstructed haplotypes is very similar to EM-based haplotypes; the analytical approach yields similar results as the simulation approach, but slightly lower.

**Table 4:** Error rate for real data scenarios.

*l_eff_*	*l*	*gene*	*Simulations using EM*		*Simulations using PHASE*		*Analytical Approach*	
			*ER_amb_*	*ER_all_*	*ER_amb_*	*ER_all_*	*ER_amb_*	*ER_all_*
1.023	2	*IL-18*	0.0000	0.0000	0.0000	0.0000	0.0000	0.0000
1.064	3	*IL-13*	0.0001±0.0008	0.0000±0.0003	0.0001±0.0008	0.0000±0.0003	0.0001	0.0000
1.149	2	*MIP1A*	0.0002±0.001	0.0001±0.0004	0.0000±0.0010	0.0001±0.0004	0.0002	0.0001
1.687	4	*INS*	0.0007±0.0019	0.0003±0.0008	0.0006±0.0017	0.0003±0.0007	0.0002	0.0001
2.313	3	*IL-6*	0.0020±0.003	0.0008±0.0015	0.0005±0.0014	0.0003±0.0007	0.0003	0.0001
2.959	3	*MCP1*	0.2560±0.046	0.048±0.01	0.2580±0.051	0.48±0.10	0.2460	0.0460
2.999	3	*TNFA*	0.456±0.166	0.01±0.0040	0.428±0.1670	0.10±0.004	0.3900	0.0090
6.384	7	*CAPN10*	0.199±0.024	0.125±0.015	0.197±0.0240	0.123±0.015	0.1870	0.1170

Values given are overall error rate (*ER_all_*) and the error rate among ambiguous subjects (*ER_amb_*)±standard deviation derived from simulations with EM-reconstruction, PHASE-reconstruction, as well as the error rate computed by the analytical approach given in Appendix S1.

### Haplotype-specific error measures: R^2^, sensitivity and specificity

**Figure 4:**
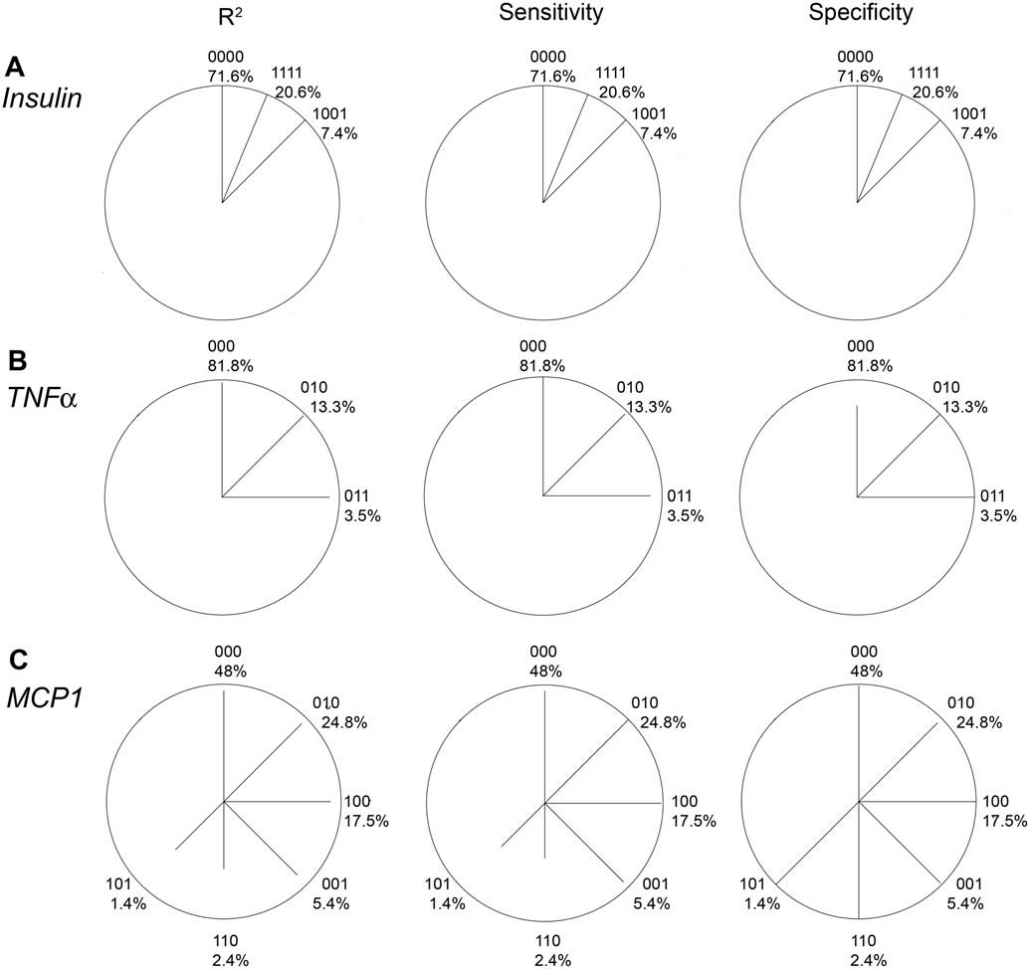
Haplotype-specific error measures: Star plots for various genes displaying R^2^, sensitivity and specificity (analytically derived) as the length of the line for each common haplotype (frequency ^3^ 1%). A line reaching the circle indicates a value of 100% (no error). Haplotypes are labelled using 0/1 coding for major/minor allele and stating the haplotype frequency. Lines are sorted clockwise by haplotype frequency beginning at the top with the most frequent haplotype. The angle between lines is given by the number of possible haplotypes, i.e. 360°/2L, where L is the number of loci. The proportion without lines thus indicates the proportion of rare or non-existing haplotypes (<1% frequency).

**Figure 5:**
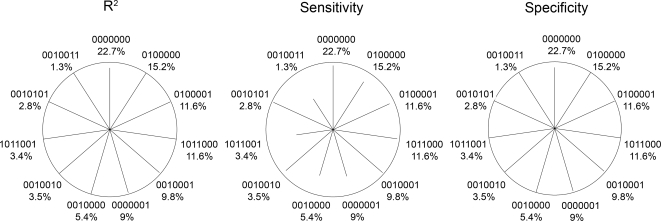
Haplotype-specific error measures: Star plots for the *CAPN10* displaying the R^2^, the sensitivity or the specificity as in Figure 4. The angle between lines is given by 360° divided by the number of frequent haplotypes (frequency ^3^ 1%) to accommodate for the large number of loci (L = 7).

We developed a star plot to summarize the haplotype-specific errors: Figure 4 and 5 show star plots for three selected genes with <5 loci and for the *CAPN10* gene involving 7 loci (*real data scenarios*). The measures were derived analytically (Appendix S1 and S2), but were very similar in the simulations. Comparing Figure 4 with Table 4 shows that high R^2^ appears together with high sensitivity, and that low error rate occurs with high R^2^ and high sensitivity.

Furthermore, it can be seen that the specificity is reduced rather for common haplotypes (e.g. 98% for haplotype 000 for *TNF*α, 97% for 0000000 of *CAPN10*). On the other side, the sensitivity is reduced rather for rare haplotypes (e.g. 101 of *MCP1*). However, there are also rare haplotypes which show almost perfect sensitivity (e.g. 0010101 for *CAPN10*).

### Impact of haplotype error on Odds Ratio estimates

**Figure 6:**
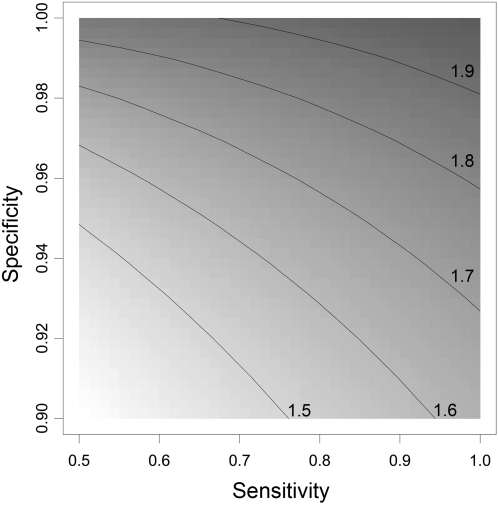
Impact of decreased sensitivity and specificity on a true odds ratio of 2.0 for a specific risk haplotype (f_h_ = 0.2) in a case-control study (500 cases, 500 controls). The shades of grey code for the observed OR with contour lines given for specific observed ORs as derived by the matrix method.

Figure 6 illustrates how the observed odds ratio (OR) for a specific risk haplotype decreases with decreasing sensitivity and specificity of reconstructing this risk haplotype, here shown for a given OR of 2 and frequency of risk haplotype of 0.2. Note the different scales for sensitivity and specificity. For example, if the risk haplotype is reconstructed with specificity 0.995 and sensitivity 0.500, an OR of 1.8 will be observed, although the true OR is 2.0. The same bias can be expected for a specificity of 0.960 and sensitivity 0.995. Therefore, only a very small decrease in specificity from 1 can result in the same bias as a high decrease in sensitivity.

## Discussion

We provide a classification of the various error measures of haplotype reconstruction error. We introduced sensitivity and specificity well-known from other areas of biomedical research to the context of haplotypes and present an analytical computational approach. We illustrated the quantity of the various error measures and their dependencies upon haplotype frequency, minor allele frequency, correlation, number of loci and ambiguity in a systematic way. Our data emphasize the dependence of the haplotype reconstruction error on the specific situation, the importance of haplotype-specific error measures and the possible impact on association analyses.

### Overall error measure based on haplotype frequencies or on individuals' haplotypes (discrepancy versus error rate)

While the discrepancy measures the error in the haplotype frequencies, the error rate depicts the error in the individual's haplotype assignment. Both measures summarize across all the different haplotypes in the sample. Fallin and Schork [13] stated that the discrepancy was small. This is supported by our data, as we observed discrepancies less than 0.005. However, we found that this observation of small discrepancies could not be transferred to individuals' haplotype error, for example the error rate.

The overall error rate is the most reported error measure [8]–[12] and indicates the percentage of subjects with either of the two haplotypes wrongly assigned. Our data showed that the overall error rate depended heavily on the specific setting: The error rate was generally increasing with decreasing correlation between the alleles and increasing ambiguity fraction. The latter also explains the increasing error rate with increasing number of loci and increasing minor allele frequency due to the enlarging proportion of subjects with ambiguous genotypes (heterozygous genotypes for at least two loci). While the error rate was small - well below 1% - in some real data examples (e.g. *IL-18*, *INS*), which indicates that 99% of subjects have perfectly reconstructed haplotypes, it was substantial in others (*CAPN10*, *TNFA*) with error rate up to 12%.

### Overall or haplotype specific error measures (error rate versus R^2^ and misclassification probabilities)

The error rate is useful as a measure to summarize across all the different haplotypes in a sample. However, an investigator is usually interested in a specific risk haplotype and in how to interpret this haplotype's association estimate. Then the question arises whether this specific haplotype is reconstructed with great error, and the error rate averaging across all haplotypes is not of much help.

A known haplotype-specific measure for haplotype reconstruction error is *R_m_^2^*, which indicates the proportion of haplotype variance explained by the genotypes. It captures the haplotype-specific error *H_m_*→H_m_*, which is the error from using the individual's expected instead of the true number of copies of a haplotype. This is a very complex error model, as it moves from the discrete space {0,1,2} to the continuous space [0,2] with the distribution of H_m_* being three-modal at 0,1, and 2. This error measure can only be applied when using the expected number of haplotypes H_m_* as explaining variables in the haplotype association analysis.

An alternative is a haplotype-specific measure for the error *H_m_*→C_m_*, that is the error from using the individual's most likely number of copies of the haplotype instead of the true number of copies. This is an error model from the discrete space {0,1,2} into the discrete space {0,1,2} and thus a classical misclassification problem, which is represented by the 3×3 misclassification matrix. This is very appealing as the full concept of misclassification is then available. When the misclassification matrix is known, methods are available to account for the error, e.g. by means of the matrix method [30] or the MC-SIMEX [23].

### Sensitivity and Specificity

As new notions of haplotype-specific error measures, we introduced the sensitivity and the specificity to the context of haplotypes and we illustrated that they complemented the R^2^ and differentiated between two reasons for haplotype reconstruction error:

Firstly, the specificity is an issue for common haplotypes: If the specificity is reduced, it is reduced rather for a common haplotype (Figure 4 and 5). This is plausible due to the fact that if any haplotype is misclassified, it is rather misclassified as a common haplotype by pure chance. Therefore, a common haplotype is more likely falsely assigned than a rare haplotype.

Secondly, the sensitivity is an issue for rare haplotypes: If the sensitivity is reduced, it is reduced more likely for a rare haplotype. For example, the rather low sensitivity of the haplotype 101 of *MCP1* (Figure 4) was due to the fact, that this haplotype most likely paired with the most common haplotype 000 given Hardy-Weinberg equilibrium (haplotype pair 101/000) and that the alternative haplotype pair 001/100 contained two rather frequent haplotypes (001 and 100 with frequencies 5.4% and 17.5%). Thus, the haplotype pair 101/000 would often be falsely reconstructed as 001/100. Generally speaking, the haplotype pair containing a rare haplotype - and thus the rare haploptype itself - is more likely falsely classified. On the other side, there are also rare haplotypes that are perfectly reconstructed, which occurs when there is no likely alternative haplotype pair. For example, the haplotype 0010101 of *CAPN10* (Figure 5) showed almost 100% sensitivity: Besides the haplotype pair consisting of this rare haplotype and the most common haplotype, 0010101/0000000, an alternative would have been 0010001/0000100 or 0010000/0000101. However, for both alternatives, none of the two haplotypes did exist with frequency >1%. Thus the probability of such a pair was negligible, the pair 0010101/0000000 was assigned with great certainty, and the rare haplotype 0010101 was very well reconstructed.

We also illustrated that sensitivity and specificity completely describe the misclassification matrix (Table 2) and thus provide the prerequisite for methods accounting for misclassification.

### Impact on Odds Ratio Estimates

We elucidated the magnitude of bias for a case-control study assuming a dominant model of the risk haplotype. The OR is substantially biased, if the haplotype is reconstructed with either small sensitivity and a specificity of 1, or a sensitivity of 1 and only a very small deviation with respect to the specificity, or with a reduction of both, sensitivity and specificity. In our real data examples, we observed a somewhat small specificity for the most common haplotype of *TNFA* and a small sensitivity for the rare *MCP1* haplotypes (Figure 4). For these haplotypes, one has to think about potentially biased estimates. For all other haplotypes, specificity is 100% and sensitivity so high, that a remarkable bias is not expected. If interest lies in a certain risk haplotype, for example haplotype 0010101 of *CAPN10* (Figure 5), one does not have to worry about biased estimates since it is reconstructed with certainty although it is rather rare.

### Star plot for haplotype-specific error measures

The quantity of reconstruction error is hard to predict intuitively as the reconstruction depends on the full constellation of the other haplotypes. To better judge whether the haplotype association estimate is biased due to substantial reconstruction error, looking at the haplotype-specific error measures would greatly enhance the knowledge about the reliability of haplotype association estimates. We have thus developed a graphical tool to comprehensively display the haplotype-specific error measures R_m_^2^, sensitivity or specificity, which is available as R-function in Appendix S2.

### Computational considerations

The analytical derivations of error rate, sensitivity and specificity complement the computational formula of *R_m_^2^* [25]. The simulations validated the analytical approach also comparing EM- versus PHASE-reconstruction. It should be noted that the error measures in the simulations included the sampling error and were thus slightly higher than the analytically derived measures, but the difference was not substantial due to sufficient sample size. Comparing EM- with the PHASE-reconstruction, we found that both methods worked equally well when applying *real data scenarios*. The *abstract scenarios*, while being useful to make extreme examples and to understand mechanisms, included situations such as the no-LD scenario under which no haplotypes should be reconstructed in the first place.

### Strengths and Limitations

We consider the classification and systematic investigation of error measures a useful guidance for researchers interested in haplotypes and haplotype association estimates. This was strengthened by applying both analytical and simulation approaches for numerous scenarios, by exemplifying the measures to real data and by utilizing the two main reconstruction methods. Finally, this is the first work investigating the sensitivity and specificity of haplotype reconstruction and illustrating their impact on haplotype association analyses.

It might be considered a limitation that we used reconstructed haplotype frequencies from real data as “true” haplotype frequencies for our *real data scenarios*. However, this is an excellent procedure to yield near-realistic haplotype distributions; the discrepancy was rather small, so that reconstructed haplotype frequencies could be assumed to approximate the true frequencies fairly well. Due to the lack of a gold standard, we can only provide an estimation of expected haplotype misclassification based on the frequencies of observed haplotypes. Levenstien at al. [31] presented a method which uses molecular haplotypes on a subset of individuals to estimate haplotype misclassification and account the Likelihood Ratio test for it in the setting of case-control studies. However, due to the absence of high throughput procedures for molecular haplotyping, this method is too time- and money-consuming in most cases. Furthermore, even haplotypes assessed in the laboratory are subject to error and can thus also not be taken as a gold standard procedure.

It might also be a limitation that we investigated an extensive, but not universal set of scenarios. For example, our scenarios were restricted to haplotypes across 2 to 7 loci, while in practice there are up to 20 loci. The restriction was made for the sake of limiting the complexity, hypothesizing that the general findings can be transferred to longer haplotypes. Finally, we assumed Hardy-Weinberg equilibrium (HWE) for the haplotype pairs and did not evaluate the impact of violation of this assumption as other work has already focused on this issue (e.g. [10]).

It might be argued that inferring individual haplotypes at all is not an appropriate approach and thus there is no reason for looking at haplotype uncertainties. In the light of numerous methods, which have been developed to simultaneously estimate haplotype probabilities together with association estimates, this argument seems to have a big relevance. These methods do not infer individual haplotypes but use the expected values of haplotypes given the observed genotypes in association analysis within a likelihood framework [16]–[18], [20] or with estimating equations [32]. These approaches involve a very complex error model from the true haplotype as a trichotomous variable into the continuous space of the expected number of haplotype copies. They are often limited to case-control studies, can not incorporate environmental variables or assume additive effects, which is often not the case [33]. Individually inferred haplotypes, on the contrary, can easily be incorporated into generalized linear models (GLM), which provides wide flexibility in the modelling of underlying inheritance assumptions, the study type, the type of outcome variable and gene-environment interactions. Due to the ease of computation in each standard statistical software, this method is quite popular in practice. Furthermore, if some haplotypes can be expected to be inferred correctly or with only small error with respect to sensitivity and specificity as in the example of the *CAPN10* haplotype mentioned, analyses based on individually inferred haplotype are most powerful. However, association estimates can be biased substantially, if high haplotype misclassification is involved [34], [35]. In these cases, information of the misclassification probabilities can be used to correct association estimates (e.g. using the MC-SIMEX method [23]) and still stay in the flexible GLM framework.

### Summary and Conclusion

In this work, we provide a classification and systematic quantification of haplotype reconstruction error measures. Our results underscore the value of haplotype-specific error measures. We introduce the well-known and easily communicated concept of sensitivity and specificity to the context of haplotypes. We provide an analytical computational approach, and a graphical tool for a summary presentation, which allows to routinely quantify sensitivity and specificity next to haplotype frequencies and haplotype association estimates to provide a sense of certainty into the haplotype reconstruction, especially if interest lies in one specific risk haplotype.

This has the advantage that then the misclassification matrix is known thus providing the necessary prerequisite for methods to account for misclassification for example via the Matrix Method or the MC-SIMEX. We conclude that haplotype association analyses can greatly benefit from quantifying haplotype-specific error on a routine basis.

## Supporting Information

Appendix S1 Analytical approach to quantify the error rate, sensitivity and specificity
(0.12 MB DOC)

Appendix S2 Function ‘Sensitivity’ calculates sensitivity, specificity and efficiency for each haplotype and Function ‘Starplot’ draws sensitivity, specificity or R-square-values for each haplotype (R-programs)
(0.01 MB TXT)
