# Supplementary material for: Haplotype Reconstruction Error as a Classical Misclassification Problem: Introducing Sensitivity and Specificity as Error Measures
Source: PLoS One. 2008 Mar 26;3(3):e1853. doi: 10.1371/journal.pone.0001853 (PMC2267219; doi:10.1371/journal.pone.0001853)
Supplement: Appendix S1 — Analytical approach to quantify the error rate, sensitivity and specificity (0.12 MB DOC) [file pone.0001853.s001.doc]

# Appendix S1:

Analytical Approach to quantify the error rate, sensitivity and specificity

**Error rate**

The probability that the haplotype pair of individual *i* is correctly inferred, $P(H_{i}=C_{i}*)$, can be written as

$\sum_{\eta} P(C_{i}*=\eta,H_{i}=\eta)=\sum_{\eta} P(H_{i}=\eta)P(C_{i}*=\eta|H_{i}=\eta)$ (1).

We can write $P(C_{i}*=\eta|H_{i}=\eta)=P(C_{i}*=\eta|H_{i}=\eta,G_{i}=\gamma)P(G_{i}=y|H_{i}=\eta)$. Since $C_{i}*$ depends only on *G_i_*, the first factor reduces to $P(C_{i}*=\eta|G_{i}=\gamma)$and the second factor equals unity since *G_i_* is uniquely defined by the haplotype pair *H_i_*. Thus, with being the sum over all possible haplotype pairs *η*, which are consistent with  γ, $\eta\sim\gamma$, (1) can be restated as

$\sum_{\gamma} \sum_{\eta:\eta\sim\gamma} P(H_{i}=\eta)P(C_{i}*=\eta|G_{i}=\gamma)$ (2).

The $\eta$ among all $\eta\sim\gamma$ that maximize $P(H_{i}=\eta|G_{i}=\gamma)$ will be denoted by $\eta_{best}(\gamma):=\mathop{\mathrm{argmax}}\limits_{\eta\text{:}\eta\text{ \textasciitilde}\gamma\text{ }} P(H_{i}=\eta|G_{i}=\gamma)$, which is assigned as the most likely haplotype pair to $C_{i}*$ given the genotype. Thus, $P(C_{i}*=\eta_{best}(\gamma)|G_{i}=\gamma)=1$ and $P(C_{i}*=\eta|G_{i}=\gamma)=0$ for other $\eta:\eta\sim\gamma$, and (2) reduces to

$\sum_{\gamma} P(H_{i}=\eta_{best}(\gamma))$ (3).

or written alternatively as $\sum_{\gamma} \max_{\eta:\eta\sim\gamma} P(H_{i}=\eta|G_{i}=\gamma)$. With $f_{\eta}$denoting the probability of a subject having haplotype pair $\eta$, this becomes $\sum_{\gamma} \max_{\eta:\eta\sim\gamma} f_{\eta}$. $f_{\eta}$is given by $f_{\eta}$=$2f_{j}f_{k}$ for $j\neq k$ or $f_{\eta}$=$(f_{j})^{2}$ otherwise, when $\eta$ depicts the haplotype pair h_j_/h_k_. If the reconstructed haplotype frequencies, $\hat{f}_{\eta}*$, can be assumed to approximate the true haplotype frequencies (i.e. the haplotype-specific discrepancy is small), an approximation of the error rate is thus given by

**Sensitivity and specificity**

For each haplotype *h_m_*, *m*=1,…,*M*, the sensitivity and the specificity are defined as $Sn_{m}=P(C_{im}*>0|H_{im}>0)$ and $Sp_{m}=P(C_{im}*=0|H_{im}=0)$, respectively. The sensitivity can be rewritten as

$Sn_{m}=\frac{P(C_{im}*>0,H_{im}>0)}{P(H_{im}>0)}$ (4).

The numerator of (4) can be computed as $\sum_{\eta,\xi:\eta_{m}>0,\xi_{m}>0} P(C_{i}*=\eta,H_{i}=\xi)$, which we separate into two sums $\sum_{\eta=\xi:\eta_{m}>0,\xi_{m}>0} P(C_{i}*=\eta,H_{i}=\xi)+\sum_{\eta\neq\xi:\eta_{m}>0,\xi_{m}>0} P(C_{i}*=\eta,H_{i}=\xi)$, and due to $\sum_{\eta\neq\xi:\eta_{m}>0,\xi_{m}>0} P(C_{i}*=\eta,H_{i}=\xi)$ being zero, we yield

$\sum_{\eta:\eta_{m}>0} P(C_{i}*=\eta,H_{i}=\eta)$ (5).

With Bayes’ Formula, we get $\sum_{\eta:\eta_{m}>0} P(H_{i}=\eta)P(C_{i}*=\eta|H_{i}=\eta)$. We then apply the same deduction as from (1) to (3), but restricting the sum to a sum of γ, which yield $\eta_{best}(\gamma)$ such that the *m*th component of $\eta_{best}(\gamma)$ is > 0. Then (5) can be restated as

$\sum_{\gamma: \eta_{best}(\gamma)_{m}>0} P(H_{i}=\eta_{best}(\gamma))$ (6),

and the sensitivity can be derived as

$$Sn_{m}=\frac{\sum_{\gamma:\eta_{best}(\gamma)_{m}>0} P(H_{i}=\eta_{best}(\gamma))}{\sum_{\eta:\eta_{m}>0} P(H_{i}=\eta)}=\frac{\sum_{\gamma:\eta_{best}(\gamma)_{m}>0} f_{\eta_{best}}}{\sum_{\eta:\eta_{m}>0} f_{\eta}}$$

and the specificity, analogously, as

$Sp_{m}\frac{\sum_{\eta_{best}(\gamma)_{m}=0} P(H_{i}=\eta_{best}(\gamma))}{\sum_{\eta:\eta_{m}=0} P(H_{i}=\eta)}=\frac{\sum_{\eta:\eta_{best}(\gamma)_{m}=0} f_{\eta_{best}}}{\sum_{\eta:\eta_{m}=0} f_{\eta}}$.

**Computing the error rate from sensitivity and specificity**

In order to describe the error rate $1-P(H_{i}=C_{i}*)$ $=1-\sum_{\eta} P(C_{i}*=\eta,H_{i}=\eta)$ by $Sn_{m}$ and $Sp_{m}$, the sum is partitioned into and . With (4) and (5) ,we obtain $\sum_{\eta:\eta_{m}>0} P(C_{i}*=\eta,H_{i}=\eta)=Sn_{m}\sum_{\eta:\eta_{m}>0} P(H_{i}=\eta)$ and $\sum_{\eta:\eta_{m}=0} P(C_{i}*=\eta,H_{i}=\eta)=Sp_{m}\sum_{\eta:\eta_{m}=0} P(H_{i}=\eta)$, and thus

$P(H_{i}=C_{i}*)=Sp_{m}\sum_{\eta:\eta_{m}=0} P(H_{i}=\eta)+Sn_{m}\sum_{\eta:\eta_{m}>0} P(H_{i}=\eta)=Sp_{m}\sum_{\eta:\eta_{m}=0} f_{\eta}+Sn_{m}\sum_{\eta:\eta_{m}>0} f_{\eta}$.
